# The relationship between endometriosis-related pelvic pain and symptom frequency, and subjective wellbeing

**DOI:** 10.1186/s12955-019-1185-y

**Published:** 2019-07-16

**Authors:** Georgia Rush, RoseAnne Misajon, John A. Hunter, John Gardner, Kerry S. O’Brien

**Affiliations:** 1 School of Social Sciences, Monash University, Melbourne, Australia; 2 The Cairnmillar Institute, Melbourne, Australia; 3 Psychology Department, University of Otago, Dunedin, New Zealand

## Abstract

**Background:**

This exploratory study sought to establish the relationship between endometriosis-related pelvic pain, endometriosis symptom-frequency, and women’s subjective wellbeing (SWB).

**Methods:**

A purposive sample (*N* = 2061) of women with endometriosis aged between 18 and 62 years (M = 30.49 ± 7.45) completed an online questionnaire containing a measure of pelvic pain (Biberoglu & Behrman Scale; B&B), endometriosis symptom frequency, and an established measure of SWB (Personal Wellbeing Index: PWI).

**Results:**

Mean SWB total scores (58.35 ± 17.90) were considerably lower than those of women in the general population (western normative range = 70–80; mean = 76). On average, women reported moderate levels of pelvic pain (B&B mean = 5.96 ± 1.84), with a mean of 10.87 (± 4.81) endometriosis-related symptoms across the sample. Significant relationships were found between pelvic pain and SWB dimension and total scores (*r*’s = − 0.20 to − 0.43, all p’s < .001), and significant small to medium associations between symptom frequency and all but one of the dimensions of SWB (*r*’s = − 0.12 to − 0.23, all p’s < .007). In multivariate regression models accounting for age and delay in diagnosis, higher levels of pelvic pain were significantly associated with lower SWB scores across all eight dimensions of the PWI and total score (all p’s < .002). Greater symptom frequency was significantly associated with lower levels of SWB for the dimensions of health, future security, life as a whole, and total scores (all p’s < .002).

**Conclusions:**

SWB was lower in women with endometriosis than SWB in women from the general population, and endometriosis related symptoms and pelvic pain explain significant proportions of the unique variance in women’s SWB scores. Psychosocial support is needed for women dealing with endometriosis-related symptoms and pain in order to improve their wellbeing and quality of life.

## Background

Endometriosis is a chronic condition affecting approximately 1 in 10 women of reproductive age. Characterised by endometrial-like tissue outside the uterus, endometriosis is associated with a host of negative symptoms including pelvic pain, pain during menstruation, pain during and after sexual intercourse, and bleeding in the bladder [1]. Despite the potential for endometriosis-related symptoms to negatively affect the subjective wellbeing (SWB) of women, there is a paucity of research on SWB in women with endometriosis.

SWB is a multi-faceted construct comprised of cognitive and affective components, and broadly defined as an individual’s evaluation of satisfaction with their life [2, 3]. While SWB is typically high in general population samples, SWB appears significantly reduced for many chronic medical conditions such as HIV, arthritis, diabetes, and psoriasis [4]. Importantly, lower levels of SWB have been found to be associated with a range of negative psychological and social outcomes such as suicide ideation, depression, anxiety, and social isolation [5–7]. Although sparse, research examining SWB in women with endometriosis suggests SWB is lower than in population samples, and lower than a number of other chronic conditions, including diabetes [4].

The paucity of research on SWB in women with endometriosis is exacerbated by an absence of research seeking to establish what factors may be related to the reduced levels of SWB in women with endometriosis. For example, there has been no research examining whether frequency of endometriosis symptoms is related to SWB, and little work examining relationships between endometriosis-related pain and SWB, despite theoretical arguments for such work [8]. The mental burden of managing the symptoms and associated pain of endometriosis should logically impact SWB, and SWB is a known predictor of health and mortality [9]. Research on this issue is important because of the high prevalence of endometriosis, low levels of SWB, and the known association between SWB and depression, anxiety, and suicide ideation [5–7].

The present exploratory study addresses this gap in the research literature on SWB in women with endometriosis [10] by examining the relationship between endometriosis-related pelvic pain and symptom frequency and multi-dimensional SWB in women with endometriosis. In doing so, the research seeks to inform health professionals on the potential needs for psychosocial support for women with endometriosis.

## Methods

### Participants

Women (*N* = 2061) with a formal diagnosis of endometriosis were recruited to the study via peak Endometriosis organisations in Australia and New Zealand, and via social media advertising and associated word-of mouth snowballing. The present sample is a part of a larger ongoing study on endometriosis and polycystic ovary syndrome. Table 1 displays participant characteristics in detail.

**Table 1:** Participant characteristics

Characteristics		
Age Groups	Frequency	Percent
Aged 18–25	623	30.4%
Aged 26–35	978	47.7%
Aged 36–62	451	22.0%
Health Specifics	Mean & SD	Range
Age of diagnosis	24.65 ± 6.53	10–49
Age of onset of symptoms	16.85 ± 5.54	8–45
Delay in diagnosis (years)	7.78 ± 6.20	0–40
Years with endometriosis	5.54 ± 5.88	0–36
Number of surgeries	2.36 ± 2.77	0–50
Age of diagnosis	24.65 ± 6.53	10–49
Age of onset of symptoms	16.85 ± 5.54	8–45
Type of surgery	Frequency	Percent
Laparoscopy	1622	78.7%
Hysterectomy	138	6.7%
Other	138	6.7%
Average length of cycle	Frequency	Percent
0–21 days	345	17.1%
21–34 days	1432	71.2%
> 35 days	235	11.7%
Average length of period	Frequency	Percent
0–3 days	164	8.1%
3–6 days	1118	55.5%
> 7 days	734	36.4%
Stage of endometriosis	Frequency	Percent
Stage 1	102	5.3%
Stage 2	184	9.5%
Stage 3	297	15.4%
Stage 4	543	28.2%
Unsure	802	41.6%
Type of endometriosis	Frequency	Percent
Ovarian	1021	49.5%
Rectovaginal	666	32.3%
Bladder	521	25.3%
Umbilical	93	4.5%
Other	529	25.7%
Unsure	649	31.5%

Type of endometriosis and type of surgery are not mutually exclusive. Differences in N’s across characteristics represents non-response to specific items

### Materials

After demographic and health information was collected, including age, age of onset of symptoms and delay in diagnosis (age of onset of symptoms - age of diagnosis = delay in diagnosis) participants were presented with a list of common endometriosis symptoms and asked to select which of these they currently experience. These responses were collated to form the endometriosis symptom frequency variable.

The Biberoglu and Behrman Scale (B&B) was used to assess three pain symptoms; pelvic pain, dysmenorrhea (painful menstruation), and dyspareunia (painful sexual intercourse). The B&B scale specifically asks participants to indicate whether the various symptoms are absent = 0, mild = 1, moderate = 2, or severe = 3. Scores for each pain symptom are combined into a single B&B pelvic pain score [11].

The Personal Wellbeing Index (PWI), is a seven-item measure of SWB scored on a scale from 0 = completely-dissatisfied to 10 = completely-satisfied. The PWI has excellent psychometric properties [12, 13], and assesses seven dimensions of SWB; standard of living, achieving in life, personal relationships, safety, health, community-connectedness, future security, as well as a whole of life satisfaction item [12, 14]. Individual SWB dimension and SWB total scores are typically calculated for evaluations and analysis.

### Procedure

Participants were recruited to the study via peak organisation promotion, associated websites, and via social media and snowballing/word of mouth. A link to the study was provided to participants via these platforms, and the questionnaire was hosted by the Qualtrics research platform. Participants were informed about the true nature of the study and they provided tacit consent by participating. The survey was confidential and anonymised prior to analysis. Ethical consent for the study was obtained from Monash University’s Human Ethics Review Board.

### Data analysis

Pearson’s correlation coefficients were calculated to establish bivariate relationships between pelvic pain, symptom frequency, and SWB dimension and total scores (PWI scales). Multivariate linear regression models tested multivariate relationships between the pelvic pain, symptom frequency, and SWB dimension and total scores. All models accounted for age and delay in diagnosis. To increase robustness of the standard errors, bootstrapping with 2000 resamples was used for regression models. Bonferroni adjustments to deal with multiple testing resulted in the p-critical value being set at *p* < .002.

## Results

### Descriptive analyses

Table 2 reports participants' SWB scores across all dimensions and total score. Participants' SWB scores were below population normative ranges in all dimensions (total SWB western normative range = 70–80). In this study, participants' health SWB was significantly lower than all other dimensions, and subjective safety was the highest rated dimension.

**Table 2:** Means, SDs, and ranges for subjective wellbeing (PWI) dimension and total scores

PWI	Mean	Range	SD
*N* = 2039			
Life as a whole	6.02	0–10	2.12
Standard of living	6.47	0–10	2.19
Health	3.89	0–10	2.20
Achieving in life	5.59	0–10	2.32
Safety	6.94	0–10	2.28
Personal relationships	6.83	0–10	2.46
Future security	5.66	0–10	2.49
Community connectedness	5.46	0–10	2.34
PWI total score	58.35	0–100	17.90

Just over half of participants (50.1%) scored within the moderate pelvic pain range (mean pelvic pain score = 5.96 ± 1.84; range 0–9) on the B&B scale. A breakdown of scale scores showed that 41.7% of women reported severe pelvic pain, 20.4% reported severe dyspareunia (painful sexual intercourse) and 35.7% reported severe dysmenorrhea (painful periods). Only 0.4% indicated they have no endometriosis-related pelvic pain.

Participants on average reported 10.87 ± 4.81 endometriosis-related symptoms. The most common symptoms were tiredness or lack of energy (84.4%), abdominal pain (82.5%), pain before and during periods (78.9%), pelvic pain (78.8%), back pain (75.4%), and abdominal bloating (77.2%). Only 0.2% of the sample experienced no symptoms.

### Correlations

There were small to medium sized statistically significant correlations between endometriosis-related pelvic pain and SWB dimension and total scores (*r’*s = −.20 to −.43, all p’s < .001), with higher levels of pelvic pain associated with lower levels of SWB. There were small but significant relationships between higher symptom frequency and lower SWB across all SWB dimensions (*r*s = −.12 to −.23, all p’s < .001), except the personal relationship dimension (*r* = −.06, *p* > .002).

### Multivariate relationships

Multivariate regression models accounting for age and delay in diagnosis, found that endometriosis-related pelvic pain and symptom frequency explained significant proportions of the unique variance in SWB dimension and total scores (see Table 3). Higher pelvic pain scores were significantly associated with lower SWB on all dimension and total scores. Endometriosis-symptom frequency was only associated with health, future security, life as a whole, and total SWB scores.

**Table 3:** Significant standardised beta’s (β) for associations between pelvic pain (B&B), symptom frequency, and SWB dimension and total scores. F-values are for full models accounting for age and delay in diagnosis. Adjusted *R*^2^ scores represent the amount of unique variance in SWB explained by pelvic pain and symptom frequency

SWB Dimensions	F-value	Independent variables	β	P	*R*^2^ change
Life as a whole	40.75	B&B	−.25	*P* < .000	.09
		Frequency	−.09	*P* = .002	
Standard of living	34.18	B&B	−.24	*P* < .000	.07
Health	96.59	B&B	−.36	*P* < .000	.18
		Frequency	−.12	*P* < .000	
Personal relationships	17.49	B&B	−.21	*P* < .000	.04
Achieving in life	35.54	B&B	−.24	*P* < .000	.08
Safety	26.16	B&B	−.21	*P* < .000	.06
Future security	38.51	B&B	−.23	*P* < .000	.08
		Frequency	−.10	*P* < .000	
Community connectedness	30.66	B&B	−.23	*P* < .000	.07
PWI Total	62.14	B&B	−.32	*P* < .000	.13
		Frequency	−.09	*P* = .002	

Bonferroni adjusted level of significance is set at *p* ≤ .002. All VIF’s ≤ 1.7

## Discussion

This exploratory study is the first to examine the relationship between endometriosis symptom frequency and SWB in women. It also addresses a paucity in research on SWB in women with endometriosis more generally. The present study found significant moderate bivariate relationships between pelvic pain and all dimensions of subjective well-being, and small but significant correlations between symptom frequency and all dimensions of subjective well-being. In multivariate analysis, accounting for age and delay in diagnosis, there were significant multivariate relationships between endometriosis-related pelvic pain, symptom frequency, and SWB. Multivariate regression models explained between 5 and 18% of the unique variance in SWB scores, suggesting that other factors beyond pain and symptomology contribute to lower levels of subjective well-being.

The results from this study are consistent with previous research examining SWB in women with endometriosis [10], however, previous research has not examined relationships between pelvic pain, symptom frequency, and SWB. It is noteworthy that the participants in the present study had considerably lower SWB scores than women within the general population across six of the life domains, with health related SWB the most affected in women with endometriosis. The levels of SWB reported here are also considerably lower than those reported for many other chronic and severe diseases, including many cancers and HIV [4, 9].

There are limitations to the study. Although the purposive sample obtained for this study is large, it still may not necessarily be representative of all women with endometriosis. Our sample was recruited online through endometriosis sites and support groups, and this may have led to an overrepresentation of women with severe endometriosis who would be more engaged with support organisations and accordingly may not capture views of women whose endometriosis is milder. It is, however, reasonable to assume the pattern of relationships between endometriosis-related pelvic pain, symptom frequency, and SWB will be similar in other samples.

Although we account for age and delay in diagnosis in our analyses it is clear, given our models only explain 5–18% of the unique variance in SWB, that other unexamined factors contribute to lower SWB. Social and psychological factors such as concerns about personal relationships, fertility, and sexual activity-related pain may also contribute to women’s lower SWB. These factors need to be examined in future research.

## Conclusions

The findings highlight the negative consequences painful endometriosis symptoms have on women's wellbeing. It is important that health providers and policy makers understand the significant impact that endometriosis has on women’s well-being, and look to provide support to ameliorate the negative impact on SWB.

## Data Availability

The anonymised dataset used during the current study are available from the corresponding author.

## Abbreviations

B&B scale: Biberoglu and Behrman Scale
PWI: Personal Wellbeing Index
SWB: Subjective Wellbeing
